# Large-scale association study for structural soundness and leg locomotion traits in the pig

**DOI:** 10.1186/1297-9686-41-14

**Published:** 2009-01-21

**Authors:** Bin Fan, Suneel K Onteru, Benny E Mote, Timo Serenius, Kenneth J Stalder, Max F Rothschild

**Affiliations:** 1 Department of Animal Science and Center for Integrated Animal Genomics, Iowa State University, Ames, IA 50011, USA; 2 Key Laboratory of Agricultural Animal Genetics, Breeding and Reproduction, Ministry of Education & College of Animal Science and Technology, Huazhong Agricultural University, Wuhan, 430070, PR China

## Abstract

**Background:**

Identification and culling of replacement gilts with poor skeletal conformation and feet and leg (FL) unsoundness is an approach used to reduce sow culling and mortality rates in breeding stock. Few candidate genes related to soundness traits have been identified in the pig.

**Methods:**

In this study, 2066 commercial females were scored for 17 traits describing body conformation and FL structure, and were used for association analyses. Genotyping of 121 SNPs derived from 95 genes was implemented using Sequenom's MassARRAY system.

**Results:**

Based on the association results from single trait and principal components using mixed linear model analyses and false discovery rate testing, it was observed that *APOE, BMP8, CALCR, COL1A2, COL9A1, DKFZ, FBN1 *and *VDBP *were very highly significantly (P < 0.001) associated with body conformation traits. The genes *ALOX5, BMP8*, *CALCR, OPG*, *OXTR *and *WNT16 *were very highly significantly (P < 0.001) associated with FL structures, and *APOE, CALCR, COL1A2, GNRHR, IHH*, *MTHFR *and *WNT16 *were highly significantly (P < 0.01) associated with overall leg action. Strong linkage disequilibrium between *CALCR *and *COL1A2 *on SSC9 was detected, and haplotype -ACGACC- was highly significantly (P < 0.01) associated with overall leg action and several important FL soundness traits.

**Conclusion:**

The present findings provide a comprehensive list of candidate genes for further use in fine mapping and biological functional analyses.

## Background

The skeleton, defined as the mineralized or mineralizable tissues, forms the essential basis for body framework in higher vertebrates [1]. The skeletal system, including bone and cartilage, serves as supportive, protective and connective roles for other organs and tissues during the growth and development of individuals, and is involved in determining the body size, shape, physical fitness and leg movement. The developmental processes of skeletons are complicated and are regulated by genetic factors and their interactions with environmental factors [1,2]. In humans, abnormal development of the skeleton can lead to or be predisposing to the incidence of a series of bone related disorders, such as dwarfism, osteochondrosis, osteoporosis, osteopetrosis and osteoarthritis, which affect the normal action capability and could result in lameness in severe cases.

Feet and leg unsoundness issues are of growing concern in the swine industry. Lameness caused by feet and leg (FL) problems and osteochondrosis are considered to be crucial causes for sow culling [3-5]. Previous culling rates have been estimated to range from 10 to 40% because of unsoundness issues in young breeding stock [3,6]. According to PigCHAMP™ 2007 annual report, the average culling rate of breeding females have been 48.65%, and 20~25% of that was caused primarily by locomotion problems. .

The evaluation of FL structure soundness can be implemented using objective and subjective methods. Radiograph, macroscopical joint lesion diagnosis and histological observation, bone length and diameter measurement on specific body locations are expensive and difficult methods for objective evaluation of FL soundness [3,7-9]. The subjective approaches are usually performed by scoring the pastern posture, gait and movement conditions of leg and feet using a scale with numbers ranging between the extreme values [10-13]. Although objective evaluation measures may be more direct and accurate for FL soundness conditions, the expense and difficulty of collecting measurements on living animals limit their application in the field. The previous studies on genetic parameters demonstrated that the heritability of FL structure traits was low to moderate ranging from 0.01–0.40 [11-15]. The genetic and phenotypic correlations among most of FL traits are adverse, and some of them have correlation with overall leg locomotion. The studies also indicated that FL unsoundness was unfavorably associated with leanness and some carcass traits [12,16,17].

Due to low to moderate heritability of FL soundness traits, it may be preferable to improve these traits using marker assisted selection (MAS). A limited number of prospective chromosomal regions related to bone strength, locomotion and osteochondrosis-related traits had been identified by previous quantitative trait loci (QTL) mapping studies in the pig [7,18]. However, very few candidate genes related to structural soundness and leg locomotion have been identified in the pig thus far. Most recently, whole genome association studies on human complex diseases have provided a great number of candidate genes pertaining to bone-related disorders [19-21]. This information makes it possible to conduct candidate gene discoveries in the pig based on the findings in humans.

The purpose of this study was to identify the candidate genes associated with body conformation, FL structure soundness and leg action in the pig, focusing a high throughput multiplex single nucleotide polymorphism (SNP) genotyping technology. The findings will provide genetic factors for structural unsoundness, which can be utilized into MAS schemes to improve these traits in pigs. The study also contributes to the understanding of comparative genetic control on skeletal development between humans and pigs.

## Methods

### Animals and scoring traits

The present study was conducted on piglets (n = 2,066) entering into the commercial herds from breeding stock originating from the Newsham Choice Genetics company between October 2005 and July 2006. These animals belonged to two genetic lines; 1,000 animals were from a grandparent maternal line and the other 1,066 animals were from a parent maternal line. All of animals in these two lines were Large White × Landrace gilts but actually were derived from different sources and are now both synthetics. The evaluation of 17 traits was carried out as when each animal reached the body weight of ~90 kg. The traits consisted of six body conformation traits including body size (length, depth and width) and body shape (hip structure, rib shape and correctness of top line); five FL structure traits per leg pair, front legs (legs turned, buck knees, pastern posture, foot size and uneven toes) and rear legs (legs turned, weak/upright legs, pastern posture, foot size and uneven toes), and overall leg action. The scoring formats for traits were modified based on *PIH 101 Feet and Leg Soundness in Swine *(Guidelines for uniform swine improvement programs, distributed by National Swine Improvement Federation) and those described by van Steenbergen [13] and Serenius et al. [12]. Scoring trait criterion and the description of scores are shown in Table 1 and Additional file 1, respectively.

**Table 1:** The description of the 17 analyzed traits of body conformation, feet and leg structure and overall leg action.

		**Score**			
				
**Trait**	**Description**	**1**	**9**	**Heritability Estimate**	**Mean (SD)**
Body length	Distance from tail to scapulae viewed from side	Short	Long	0.26^a ^0.29^d^	4.79 (0.97)
Body depth	Distance from back to sternum viewed from side	Deep	Shallow	0.34^d^	4.12 (1.22)
Body width	Rump width (Butterfly shape) viewed from rear	Narrow	Wide	0.69^a ^0.25^d^	5.38 (1.22)
Top Line	Arch straightness between shoulder and rump viewed from side	Weak	High topped	0.11–0.12^d^	5.18 (0.80) 0.52 (0.63)^e^
Hip structure	Hip line and tail setting viewed from side	Level	Steep	0.18^d^	4.30 (1.75)
Rib shape	Breast width view from the horizontal	More shape	Less shape	0.26^d^	4.33 (1.59)
Front turned in/out	Front hocks turned inward/outward from each other viewed from front	Turned out	Turned in	0.09^a ^0.09–0.16^b ^0.02–0.03^c ^0.02–0.06^d^	3.95 (0.71) 1.05 (0.70)^e^
Front pastern posture	Angle of front foot viewed from side	Weak and soft	Upright	0.38^a ^0.26–0.35^b ^0.28^d^	4.51 (1.61)
Buck knee	Over at the knee of front legs viewed from side	Upright	Severe buck knees	0.36^a ^0.28^b ^0.14–0.19^c ^0.13^d^	4.65 (1.64)
Front foot size	Front foot size	Large	Small	0.16^d^	5.28 (0.92)
Front uneven toes	Even and uniform shape of front hooves	Even	Severely uneven	0.01–0.13^b ^0.00–0.05^c ^0.09^d^	2.20 (0.96)
Rear turned in/out	Rear hocks turned inward/outward from each other viewed from rear	Turned out	Turned in	0.16^a ^0.15–0.22^b ^0.14–0.17^d^	4.02 (0.76) 1.01 (0.73)^e^
Rear pastern posture	Angle of rear foot viewed from side	Weak and soft	Upright	0.12^a ^0.29–0.32^b ^0.02–0.06^c ^0.31^d^	4.25 (1.33)
Weak rear legs	Angle of rear hocks viewed from side	Weak	Upright	0.10^a ^0.02–0.10^b ^0.14^d^	4.25 (1.33) 0.94 (0.80)^e^
Rear foot size	Rear foot size	Large	Small	0.23^a ^0.13^d^	5.17 (1.02)
Rear uneven toes	Even and uniform shape of rear hooves	Even	Severely uneven	0.48^a ^0.13–0.19b 0.07–0.18^c ^0.12^d^	2.31 (1.05)
Overall leg action	Structural soundness and movement and freedom of other defects	Excellent movement	Most severe/unable to walk	0.06^c ^0.12^d^	4.59 (1.84)

^a^Dutch Landrace and Dutch Yorkshire [13].

^b^Danish Landrace and Danish Yorkshire [10].

^c^Finish Landrace and Finish Large White [11].

^d^Commercial Landrace × Large White [12].

^e^Adjusted data by subtracting 5 from the original score and taking the absolute value for each trait in this study.

The traits were independently evaluated by two experienced scorers using a 9-point scale, where 1 and 9 indicated the extreme phenotypes of the traits. The intermediate score is the most favorable for four of the scoring traits including correctness of top line, turned front legs, turned rear legs and weak/upright rear legs, and the original scores for these four traits were adjusted by subtracting 5 from the score and taking the absolute value for each animal before performing statistical analyses.

### Gene selection and SNP genotyping

Candidate genes were selected for SNP discovery. The genes are involved in skeletal pattern development, bone matrix biosynthesis, osteoclast and osteoblast differentiation, calcium and phosphorus metabolism and bone related signaling pathways. In total, 214 genes were initially chosen and among them 95 genes were successfully analyzed in the present study (Additional file 2).

Corresponding human gene sequences for exons, introns, 5'UTR and 3'UTR were retrieved from the Ensembl database , and they were blasted using the default parameters (0.01; low complexity; 100; 100; -G5-E2) against the pig genomic sequence database to obtain homologous pig sequences ; . Primers were designed through Primer 3.0 .

Ear tissue was collected from animals using the TypiFix™ ear tag from Agrobiogen (Hilgertshausen, Germany). The DNA was isolated from dry ear tissue using the DNeasy 96 Blood & Tissue Kit (Qiagen, Valencia, CA, USA). The PCR system consisted of 12.5 ng porcine genomic DNA, 1 × GoTaq PCR buffer, 0.125 mM of each dNTP, 0.25 mM of each primer and 0.25 U GoTaq DNA polymerase (Promega, Madison, WI, USA) in a 10 μl reaction volume. The PCR conditions were 94°C for 4 min, 35 cycles of 94°C for 30 sec, optimum annealing temperature (54–62°C) for 30 sec and 72°C for 30 sec, with a final extension for 5 min at 72°C using MJ-PTC 200 thermocycler (Bio-Rad Laboratories, South San Francisco, CA, USA). PCR products from the DNA of several animals with extreme phenotypes were pooled and sequenced (DNA Facility of Iowa State University, Ames, IA, USA). Five multiplexed assays for 172 SNPs were designed by means of the MassARRAY Design software and were run through Sequenom's MassARRAY system (Sequenom Inc, San Diego, CA, USA).

### Statistical analyses

The normality testing and phenotypic correlations among traits were estimated using UNIVARIATE and CORR (Pearson) procedures of the SAS software package (SAS Institute, release 9.1, Cary, NC, USA), respectively. Genotype frequency, minor allele frequency (MAF) and Hardy-Weinberg equilibrium testing were calculated with the computer program developed by our lab. Association analyses between SNPs and the traits were carried out using the MIXED procedure of the SAS package. The statistical model used in this study is as follows:

*Y*_*ijklmnop *_= *μ *+ Animal_*i *_+ Sire_*j *_+ Gilt line_*k *_+ Evaluation date_*l *_+ Scorer_*m *_+ Genotype_*n *_+ b·(Body weight_*o*_) + *e*_*ijklmnop*_

In this model, gilt line, evaluation date, scorer and marker genotype were fixed effects; sire and animal were random effects; body weight was a covariate and b is the regression coefficient. The animals with unknown sire information were considered to be derived from a different sire in order to ensure the validity of association analyses. The significance of fixed effects was determined using Type 3 tests. The raw P-values were adjusted using multiple testing, which was implemented with resampling-based false discovery rate (FDR) methods with the MULTTEST package of the R program [22], and a 20% threshold of FDR was applied to avoid false positives and consider significant SNPs. Haplotype analyses and graphical representation of linkage disequilibrium (LD) structure as measured by *r*^2 ^were performed with the Haploview software (ver. 3.32) [23]. Haplotypes were obtained for each animal using the PHASE computer program (ver. 2.1) [24]. The association analyses between different copy numbers of specific haplotypes and traits were executed using the MIXED procedure of SAS as mentioned above.

In addition, principal component analysis (PCA) was conducted with the PRINCOMP procedure of the SAS package. The first component of PCA is the mathematical combination of measurements explaining the largest amount of variability in the data, and the association analyses between the SNPs and principal components (PC1 and PC2) in this study were performed using the MIXED model as described above.

## Results

### Phenotype statistics

The basic statistics and phenotypic correlations between the analyzed traits are listed in Table 1 and Additional file 3, respectively. Apart from the four traits with intermediate values, population average values of most traits were between 4.1 and 5.3. There was no highly significant phenotypic correlation between most of the analyzed traits. Body conformation traits showed small, generally non-significant correlations with overall leg action. PCA was performed on body conformation and FL structural traits separately because of the low phenotypic correlations between the traits (Additional file 4). For body conformation traits, the cumulative proportion of the first three principal components (PC1, PC2 and PC3) reached 72%. The PC1 was mainly comprised of body depth, body width and rib shape, which explained 34% of total variation and mainly described body volume in a biological sense. The PC2 consisted primarily of hip structure and top line traits, and described side profile and the PC3 focused on body length. However, for FL structural traits and overall leg action, PC1, PC2 and PC3 accounted for 42% of total variation. The PC1 mainly included overall leg action, front pastern posture, rear pastern posture and buck knee obtained around 20% of total variation and could be considered as an indicator for leg movement evaluation. The PC2 was mostly composed of foot size per pair, describing feet defects, and the PC3 was mainly comprised of uneven toes per pair, which reflects small inner toe problems.

### Genotyping statistics

Among the 214 genes chosen in the study, 435 SNPs were detected in 146 genes and the SNPs were deposited to dbSNP of NCBI (Accession numbers: ss86352080-ss86352515). Excluding SNPs with no call, monomorphism, mistaken inheritance, MAF less than 5% and a call rate less than 85%, 119 SNPs from 95 genes were successfully genotyped by Sequenom's MassARRAY system. Detailed information including the analyzed genes, SNP types, locations and other statistics was summarized in Additional file 2.

### Association analyses for single trait

Empirical P-values for association analyses between SNPs and a single trait, and labeled SNPs representing ones that were significantly associated with the trait (at level of 1% nominal P-value and under the 20% threshold of FDR) are illustrated in Additional file 5. A total of 106 trait-marker combinations were considered to be significant according to 20% FDR criterion. The significant SNPs are listed in Additional file 6.

A total of 69 SNPs from 54 genes had at least one significant association at the P < 0.05 level. For overall leg action, which reflects both FL structural soundness and freedom from other defects affecting the gait, 20 SNPs from 15 genes were found to be significantly (P < 0.05) associated with this trait. *APOE *was very highly significantly (P < 0.001) associated and *MTHFR, GNRHR, CALCR*, *IHH *and *WNT16 *were highly significantly (P < 0.01) associated. Multiple SNPs from *CALCR *and *COL1A2 *were significantly (P < 0.05) associated with overall leg action.

For body size traits (length, depth and width), *COL9A1 *was highly significantly (P < 0.01) associated with all three traits and *APOE, CART, INSL3 *and *DKFZ *were suggestively (P < 0.1) associated with these traits. The body shape traits (top line, hip structure and rib shape) were involved in the development of long back vertebrae, ribs, hipbones and rump muscles. *FBN1 *and *BMP8 *were very highly significantly (P < 0.001) associated with top line and *COL1A2 *and *CALCR *were very highly significantly (P < 0.001) associated with hip structure.

All of the SNPs detected from *BMPR1B, CALCR *and *COL1A2 *were significantly associated (P < 0.05) with front pasterns, and both SNPs within *OPG *were highly significantly (P < 0.01) associated with front uneven toes. All SNPs within *COL1A2 *and *CALCR *were highly significantly (P < 0.01) associated with rear pasterns. In addition, it was found that *ESR2, MC4R *and *PTHR1 *were the common genes suggestively (P < 0.1) associated with front and rear legs turn in/out, and *BMPR1B, CALCR, CASR, OXTR *and *PTHR1 *were significantly (P < 0.05) associated with front and rear pasterns. In the same manner, *ADAM12, ALXO5, ALOX15, COL9A2, MATN3, NOCT1, WNT7B *and *WNT16 *were suggestively associated (P < 0.1) with front and rear foot size and *COL1A2, MEPE, OPG, PAPPA *and *PPARG *were suggestively (P < 0.1) associated with uneven toes of all legs.

### Association analyses for principal components

Among principal components of body conformation, the genes *COL9A1, DKFZ, PAPPA *and *VDBP *were very highly significantly associated (P < 0.001) with the PC1. Similarly, *CALCR, COL1A2, FBN1 *and *OXTR *had very highly significant (P < 0.001) association with PC2. The PC1 of FL traits exhibited very highly significant (P < 0.001) associations with *CALCR *and *OXTR*. The genes *ALOX5, COL9A2 *and *WNT16 *were highly significantly (P < 0.001) associated with PC2 of FL traits.

From the results of association analyses for single trait and PCs, *APOE, CALCR, COL1A2, COL9A1, DKFZ *and *VDBP *were very highly significantly (P < 0.001) associated candidate genes for body conformation traits. *ALOX5, BMP8, CALCR, COL1A2, OPG, OXTR *and *WNT16 *were very highly significantly (P < 0.001) associated with FL structure soundness traits, and *APOE, CALCR, COL1A2, GNRHR, IHH*, *MTHFR *and *WNT16 *were highly significant (P < 0.01) genes associated with overall leg action in the pig.

### Haplotype construction and association analyses of CALCR and COL1A2

**Figure 1:**
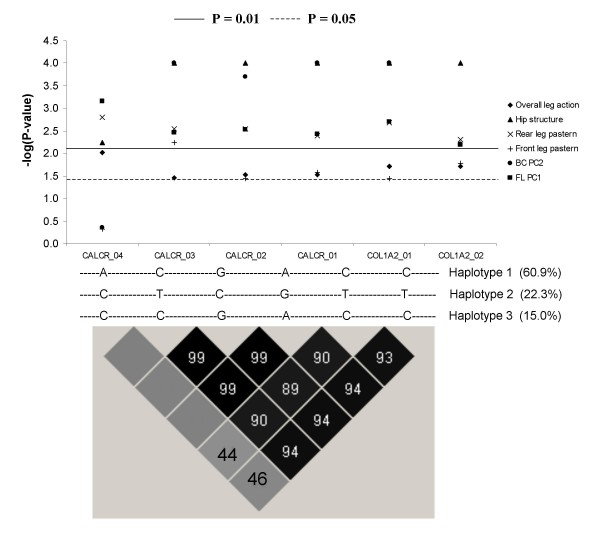
**Association analyses results of single marker and haplotype construction of SNPs within *CALCR *and *COL1A2 *in SSC9 (The *x*-axis indicates SNP ID and *y*-axis indicates -log (P-value)**. The dbSNP no. for *COL1A2*_01, *COL1A2*_02, *CALCR*_01, *CALCR*_02, *CALCR*_03 and *CALCR*_04 are ss86352086, ss86352087, ss86352109, ss86352112, ss86352113 and ss86352114, respectively. Black boxes indicate *r*^2 ^values between 0.9 and 1.0, and light boxes indicate *r*^2 ^values less than 0.80.)

All four SNPs within *CALCR *and the two SNPs within *COL1A2 *displayed a strong association with the analyzed traits, and these two genes are located adjacent to each other on SSC9, which prompted the haplotype analysis for tag SNP identification. Three major haplotypes, which accounted for 98% of alleles (Figure 1), were obtained and were shown as follows, haplotype 1, -ACGACC- (60.9%), haplotype 2, -CTCGTT- (22.3%) and haplotype 3, -CCGACC- (15%). The association results for each of these three haplotypes are shown in Additional file 7. There was a highly significant (P < 0.01) difference between individuals carrying haplotype 1 and those without haplotype 1 for traits such as overall leg action, rear pasterns, front pasterns and PC1 of FL structure. The counterpart of haplotype 1, haplotype 2, showed significant (P < 0.05) associations with the above traits. Haplotype 3 was not associated with overall leg action.

## Discussion

To our knowledge, this study is the first report on large-scale candidate gene associations with body conformation, FL soundness traits and overall leg action in the pig. The present findings provided a reliable and comprehensive list of candidate genes for further use in fine mapping and biological functional analyses.

Population structure is one of important components affecting association and linkage disequilibrium analyses. However, the significant interactions between markers and lines were only found for a very few markers in the study (data was not shown). In addition, the separate linkage disequilibrium pattern and haplotype frequencies were similar for these two lines, as well as the combination of both lines (data was not shown). Therefore, the interaction between marker and line were excluded and was not considered further in the study.

Comparative genomic approaches offer an efficient tool for candidate gene identification across closely related species. The accumulating candidate gene findings on human bone related disorders are contributing to studies in pigs. A large number of genes being considered as prospective genes for human bone related disorders were found to be associated with the analyzed traits in the pig. The genes involved in skeletal pattern development such as *WNT2, WNT16, BMP8 *and *IHH *were significantly (P < 0.05) associated with several important traits (Additional file 6). The Wnt/β-catenin pathway is critical during the development of bone and cartilage tissues. *WNT *gene family numbers such as *WNT -3a, -5a, -6, -7a, -7b, -10b *and -*11 *and Wnt associated proteins like frizzed related protein, LRP5 and β-catenin were proposed to function in bone formation [19,25]. In this study, *WNT -2, -7b, -10b *and *SFRP4 *showed significant (P < 0.05) associations with one or several body conformation and FL traits. In addition, *WNT16 *was highly significantly (P < 0.05) associated with a few of the individual traits such as overall leg action, buck knee, leg turned in/out and the principal components describing feet defects. The Wnt receptor *LRP5 *was very highly (P < 0.001) significantly associated with front foot size. These observations suggest that Wnt signaling should be analyzed further for structural traits in pigs. Bone morphogenetic proteins (BMPs) interact with their specific receptors and function in the formation of bone and skeletal patterning. The genes *BMP -2, -4, -5 *and *-7 *have been considered as inducers during the bone development processing [19,26]. Significant associations for *BMP7 *with these traits were not found in this study, but *BMP8 *was significantly (P < 0.05) associated with traits such as overall leg action and front leg pastern. This indicated that the biological functions of *BMPs *on bones may differ in pigs or exerted at different body locations. Conversely, the very highly significant (P < 0.001) association of *BMPR1B *with front pastern posture suggested that the roles it plays in the pig may be similar to humans, since the mutations in this gene were associated with brachydactyli type A2 [27].

Bone strength and geometry depend on bone matrix and bone mineral density (BMD). The genes implicating BMD variation and multiple epiphyseal dysplasias (MED) like *COL1A1, COL1A2, CO9A1, COL9A2 *and *COL9A3 *were highly suggestive in humans [19,28]. In this study, *COL1A2 *exhibited a significant (P < 0.05) association with overall leg action and other important traits. *COL9A1 *was highly significantly (P < 0.01) associated with body size traits and principal component denoting body volume. These two genes were associated with human hip osteoarthitis [29,30]. *COL2A1 *was reported to be linked to nodal osteoarthritis [29] and it was highly significantly (P < 0.01) associated with front leg pastern and front uneven toes. *COL9A2 *was significantly (P < 0.05) associated with top line and rear leg and feet soundness traits in the pig and it was related to degenerative lumbar spinal stenosis [31] and inter-vertebral disc disease [32] in humans. These results showed that further studies on these candidate genes, including additional SNP discovery and haplotype analyses, are worthwhile.

The genes affecting the functions of bone cells such as *OPG, RANKL, CALCR *and *OXTR *exhibited significant association with the analyzed traits in this study (Additional file 6). Both *RANKL *and *OPG *are important regulators of bone remodeling, and play essential roles during the osteoclastogenesis and activation of osteoclast [19,33]. In this study, one of the two SNPs from *OPG *and *RANKL *was associated with certain traits while the other SNP for each gene showed association with different traits. This implied that the SNPs might be derived from different LD blocks or they have pleiotropic effects. *CALCR *encodes calcitonin receptor, a 7-transmembrane receptor located on the surface of osteoclasts. Calcitonin activates calcitonin receptor which stimulates adenylate cyclase and leads to the inhibition of osteoclastic bone resorption. The polymorphisms of *CALCR *were related to human BMD and played a role during the pathogenesis of osteoporosis [34,35]. Strong associations of multiple SNPs located in intron 9 and 3'UTR with overall leg action and several FL soundness traits suggested that *CALCR *had significant effects on pig structural soundness and locomotion. Oxytocin receptor mediates oxytocin action through G proteins and activates a phosphatidylinositol- calcium second messenger system. A synonymous mutation in exon 3 of *OXTR *was highly significantly (P < 0.01) associated with front and rear leg pasterns and two principal components demonstrating body side profile and leg movement. Hittmeier et al. [36] found increased *OXTR *mRNA expression in bone marrow cells in pigs fed with phosphorus (P) deficient diet, and speculated that *OXTR *might affect bone growth and turnover through controlling P utilization and *PGE2 *synthesis.

The genes related to fat metabolism such as *APOE, CART, PPARG, ALOX5 *and *ALOX15 *were significantly (P < 0.01) associated with body conformation and FL traits (Additional file 6). The connections between human osteoporosis and obesity are being explored in humans. It was also reported that animals with increased fatness usually have positive FL soundness and leg action [11,17]. Apolipoprotein E is responsible for accumulating excess fat in adipose tissue [37] and adequate lipid content triggers the synthesis and secretion of leptin from adipose tissue into circulation. Leptin further acts on the hypothalamus and releases cocaine and amphetamine regulated transcript (CART) protein that inhibits bone resorption thus promoting bone strength [38,39]. Whereas, *PPARG *negatively regulates osteoblast differentiation of bone marrow stromal cells and positively promotes adipogenesis resulting in bone loss [40]. Our studies primarily gave clues that a leptin mediated neuroendocrine bone remodeling may play a key role for different levels of FL structure and body conformation traits in pigs.

Earlier QTL mapping studies on FL, leg action and osteochondrosis traits in pigs uncovered the most interesting regions and more than five QTLs were mapped on SSC1, 5, 7, 13 and 16 [7,18]. From this study, a number of interesting genes were identified on the above chromosomal regions. For instance QTLs related to FL scores for front and rear legs were mapped between microsatellite *CGA *and *S0082 *and a QTL related to front legs from side-view was mapped around *SW974 *on SSC1, where both *COL9A1 *and *ESR2 *are located. On SSC13, eight QTLs related to FL and gait traits were discovered by different researchers. The genes *PTHR1*, *PPARG*, *OXTR *and *CASR *with significant association seen in this study were between *S0068 *and *SW344 *on SSC13 where most of the QTL were mapped. The study also revealed that several prospect genes were not located in the putative chromosomal regions. For instance, the very highly significantly (P < 0.001) associated genes such as *CALCR *and *COL1A2 *were on SSC9, where only two QTL were detected. Our findings offered more valuable information for candidate genes selection in addition to those revealed by QTL studies.

Strong and highly significant (*r*^2 ^> 0.8) LD between *CALCR *and *COL1A2 *was detected. Xiong et al. [35] detected five LD blocks comprising of 27 SNPs in human *CALCR *gene, and found one SNP within 3'UTR was significantly associated with BMD and osteoporosis in the hip. It was suggested that 3'UTR of *CALCR *might be the most important region for SNP identification. The absence of haplotype 1 in pigs and the presence of its counterpart haplotype 2 favored overall leg action, but was not related to FL soundness traits such as front leg pasterns and rear leg pasterns. The contradictory results might result from the negative phenotypic correlation between overall leg action and these FL traits or the SNPs were located in different LD blocks. More SNPs within *CALCR *and *COL1A2 *are needed for further analysis.

Worth noting however, is we did not observe very highly significant (P < 0.001) association of *VDR *and *LRP5 *with body conformation, FL soundness traits and overall leg action, even though these two had been considered as important candidate genes for human bone disorders [19-21]. The reasons might be that a causative SNP has yet to be detected in the current study or they have different biological roles on the traits in the pig. In future work, an in depth-scan of SNPs and haplotype analyses are necessary for confirming the significant genes proposed by this study. Studies on interactions between genes and environments are also needed to better understand the genetic regulation mechanisms on skeleton development and the related disorders in pigs.

## Conclusion

Feet and leg unsoundness issues have become a growing problem in the swine industry, but few candidate genes related to leg soundness traits have been identified to date. Results from our study provided a reliable and comprehensive list of candidate genes associated with body conformation, FL soundness traits and overall leg action in the pig. The genes *ALOX5, BMP8*, *CALCR, OPG*, *OXTR *and *WNT16 *were very highly significantly associated with FL structures, and *APOE, CALCR, COL1A2, GNRHR, IHH*, *MTHFR *and *WNT16 *were highly significantly associated with overall leg action. Two genes *CALCR *and *COL1A2 *on SSC9 were in strong linkage disequilibrium, and one haplotype -ACGACC- was highly significantly associated with overall leg action and several important FL soundness traits. These findings motivate future studies in fine mapping and biological functional analyses to verify the effects of these genes.

## Abbreviations

BMD: bone mineral density; FDR: false discovery rate; FL: feet and leg; LD: linkage disequilibrium; MAF: minor allele frequency; MAS: marker assisted selection; PCA: principal component analysis; QTL: quantitative trait loci; SNP: single nucleotide polymorphism; UTR: un-translated region

## Competing interests

The authors declare that they have no competing interests.

## Authors' contributions

BF carried out the SNP discovery, genotyping and data analysis, and drafted the manuscript. SKO participated in the SNP discovery, genotyping and data analysis and manuscript preparation. BEM, TS and KJS carried out the traits scoring on field and data collection. MFR conceived the study, and participated in its design and coordination and helped to draft the manuscript. All authors read and approved the final manuscript.

## Supplementary Material

Additional File 1 **Appendix One. **The criteria for the scoring of the analyzed traits in the study. Click here for file

Additional File 2 **Table Two.** The characteristics of the analyzed SNPs (SNPs were sorted by their chromosomal locations). Click here for file

Additional File 3 **Appendix Two.** The phenotypic correlations between the 17 analyzed traits of body conformation, feet and leg structure and overall leg action. Click here for file

Additional File 4 **Appendix Three**. The eigenvalues and eigenvectors of principal component (PC) analysis on the 17 analyzed traits. Click here for file

Additional File 5 **Appendix Four**. Association analyses results of single SNP markers with body conformation, feet and leg structure traits and principal factors in two commercially available breeding female lines (The *x*-axis indicates individual SNPs distributed along with pig chromosomes and *y*-axis indicates -log (P-value). SNPs with P < 0.001 and being under the 20% threshold of FDR are labeled. a) overall leg action; b) body depth; c) body length; d) body width; e) hip structure; f) rib shape; g) top line; h) front leg buck knee; i) front foot size; j) front pastern posture; k) front leg turned in/out; l) front uneven toes; m) rear foot size; n) rear pastern posture; o) rear leg turned in/out; p) rear uneven toes; q) rear weak leg; r) PC1 of body conformation traits; s) PC2 of body conformation traits; t) PC1 of feet and leg structure; u) PC2 of feet and leg structure). Click here for file

Additional File 6 **Table Three. **The list of significant SNPs for the 17 analyzed traits of body conformation, feet and leg structure and overall leg action. Click here for file

Additional File 7 **Table Four**. The association analyses between putative haplotypes of *CALCR *and *COL1A2 *in SSC9, and the individual traits as well as principal components. Click here for file
