# Supplementary figures and images for: Large-scale association study for structural soundness and leg locomotion traits in the pig
Source: Genet Sel Evol. 2009 Jan 21;41(1):14. doi: 10.1186/1297-9686-41-14 (PMC2657774; doi:10.1186/1297-9686-41-14)

## Slide 1
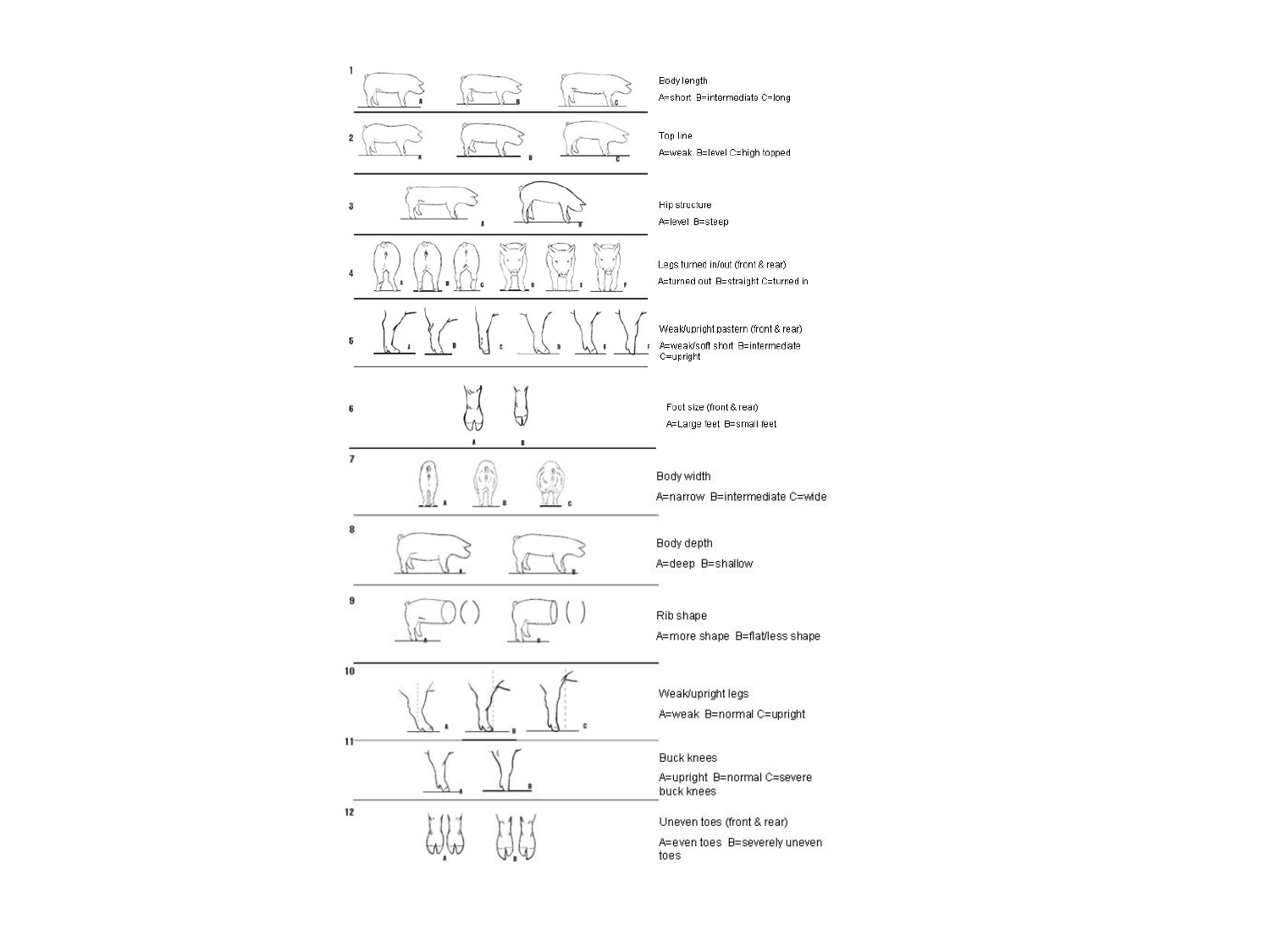

Supplement: Additional File 1 — Appendix One. The criteria for the scoring of the analyzed traits in the study. [file 1297-9686-41-14-S1.ppt]

## Slide 1
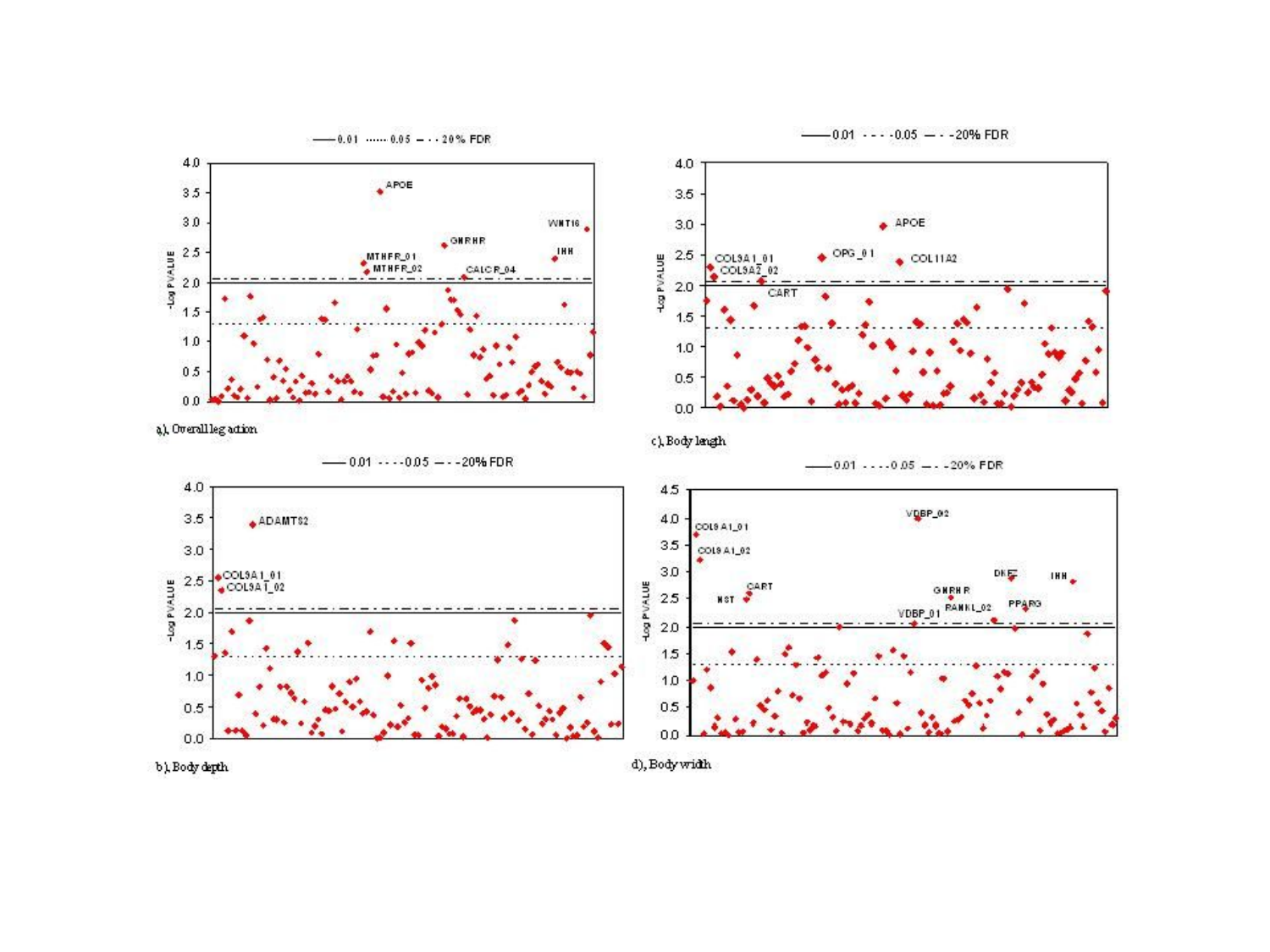

## Slide 2
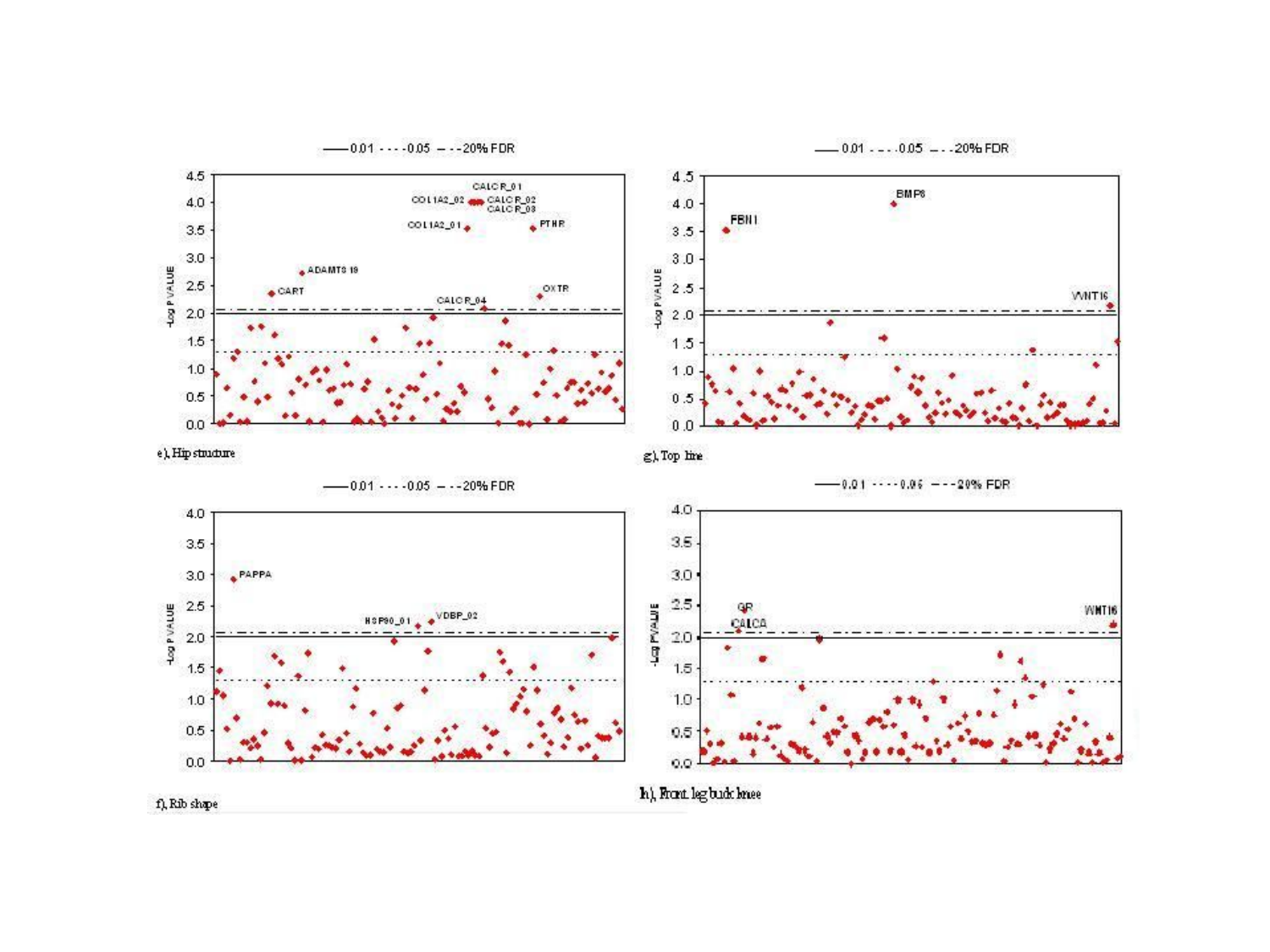

## Slide 3
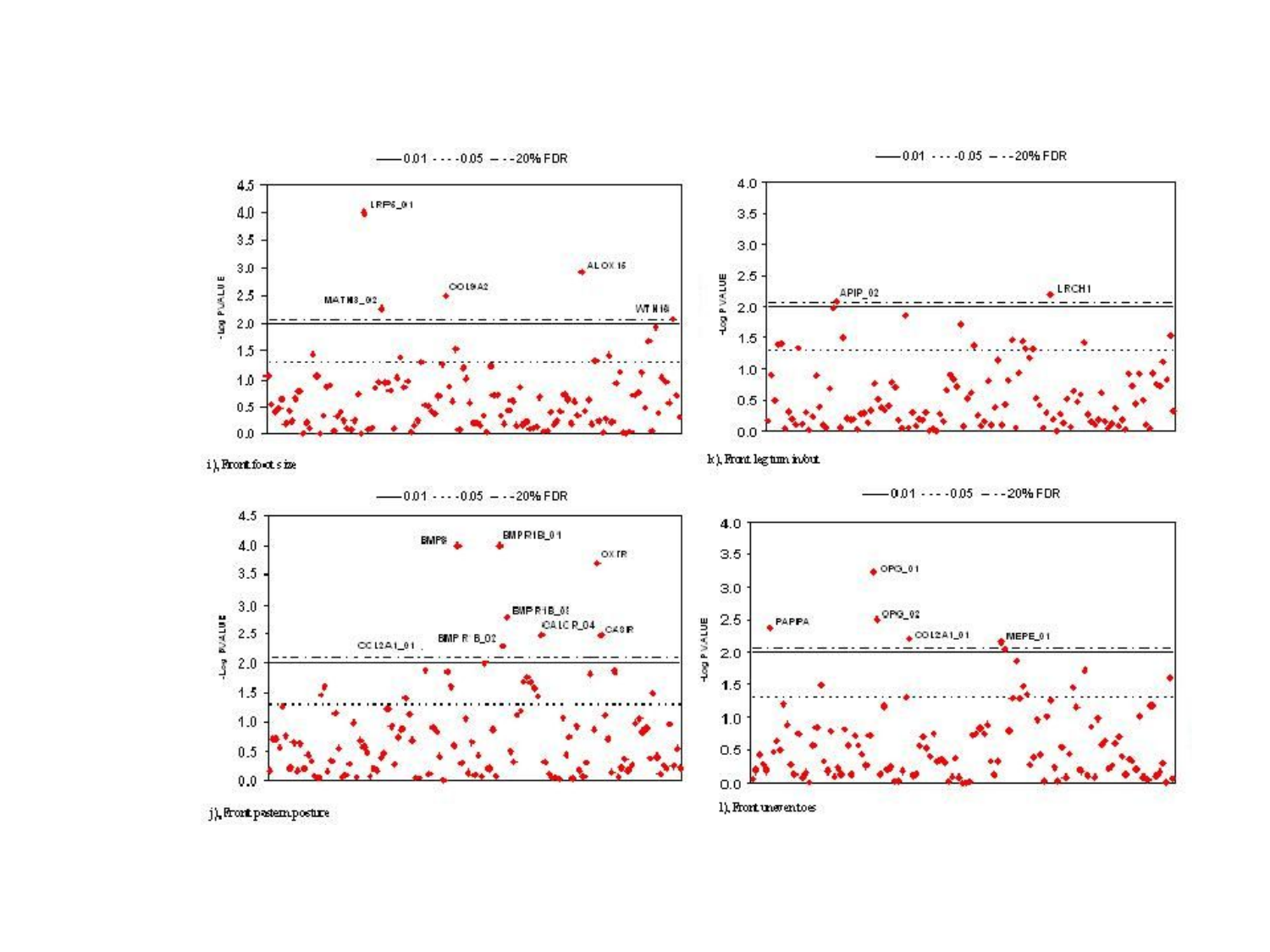

## Slide 4
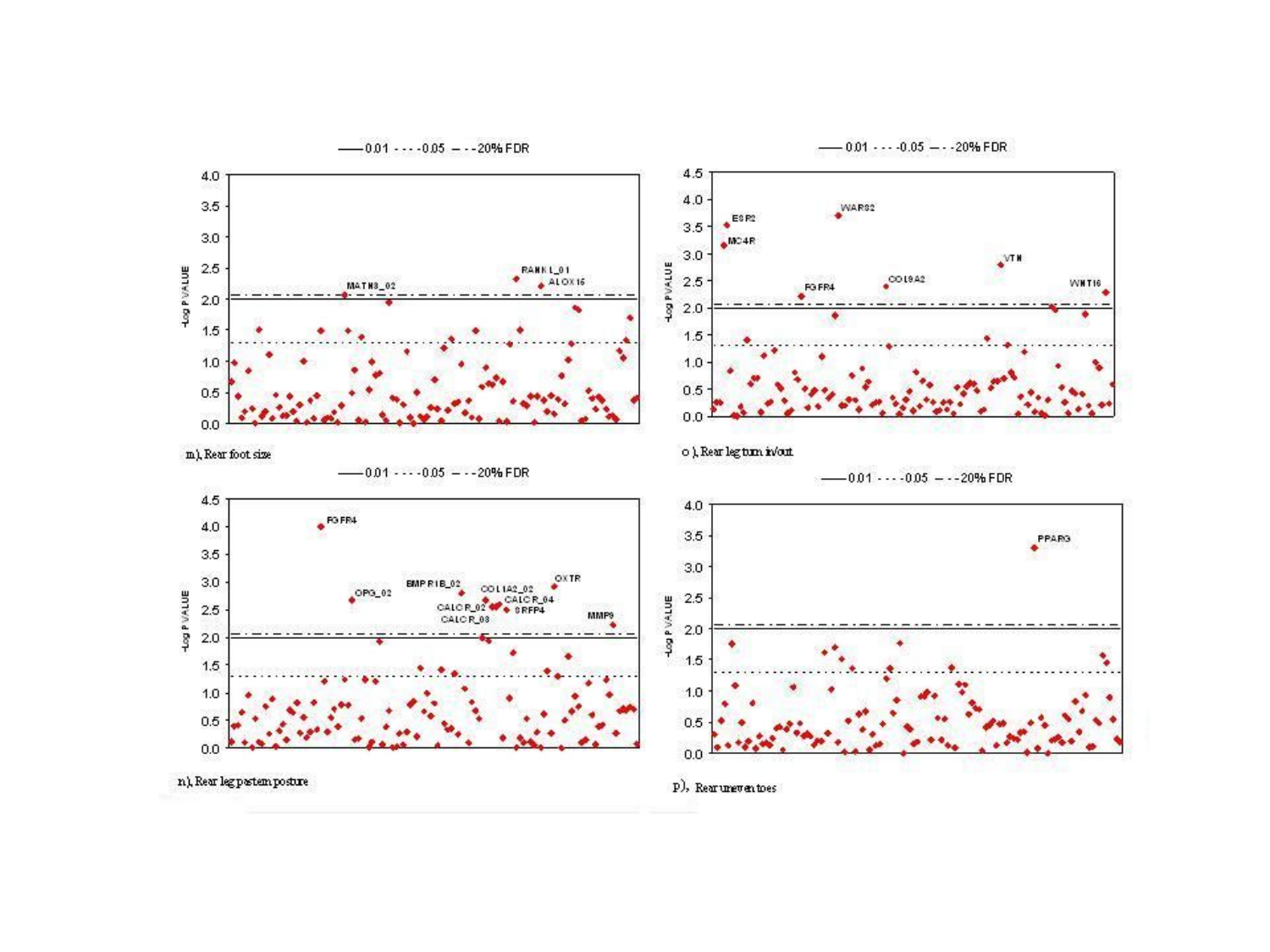

## Slide 5
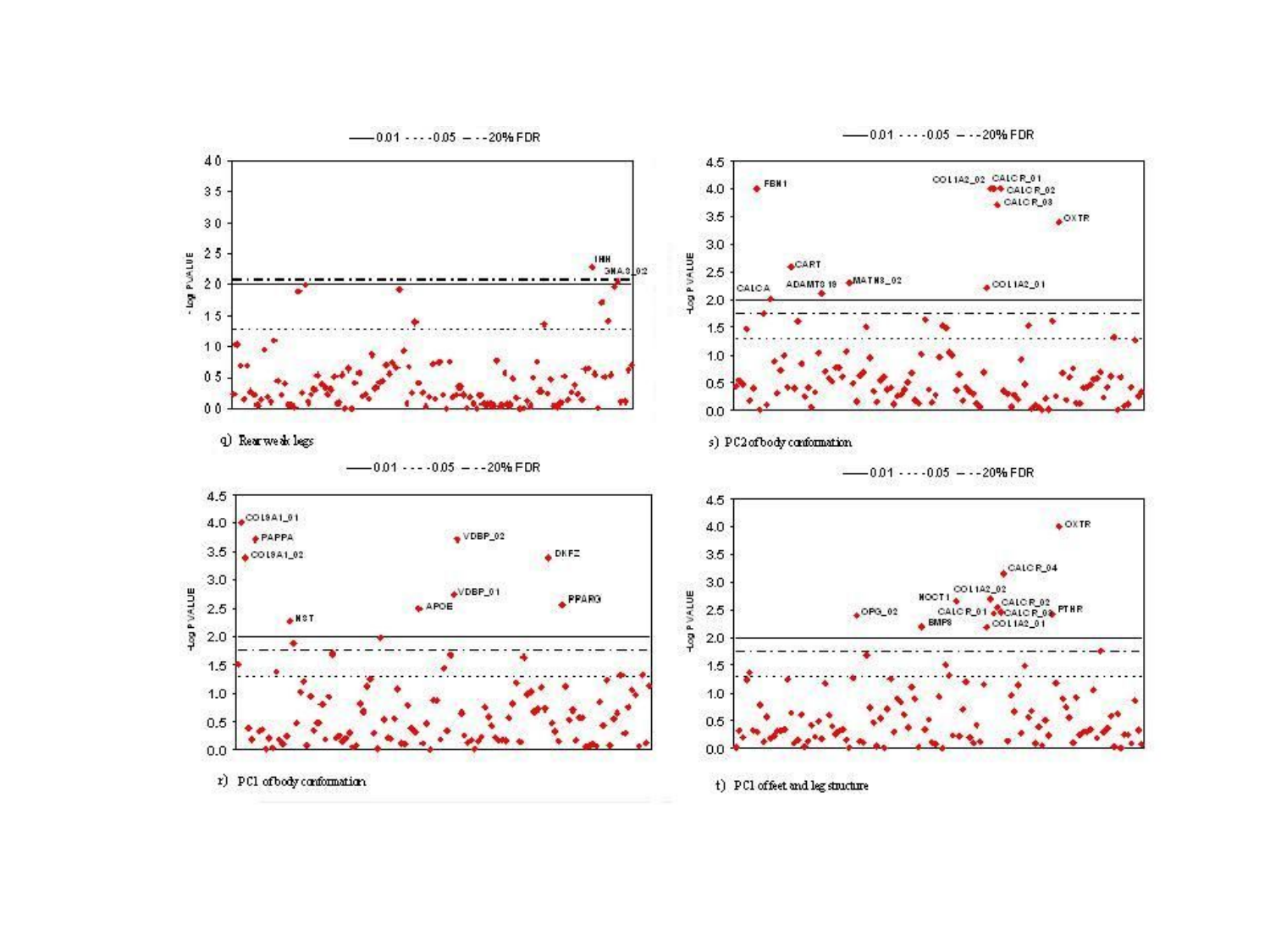

## Slide 6
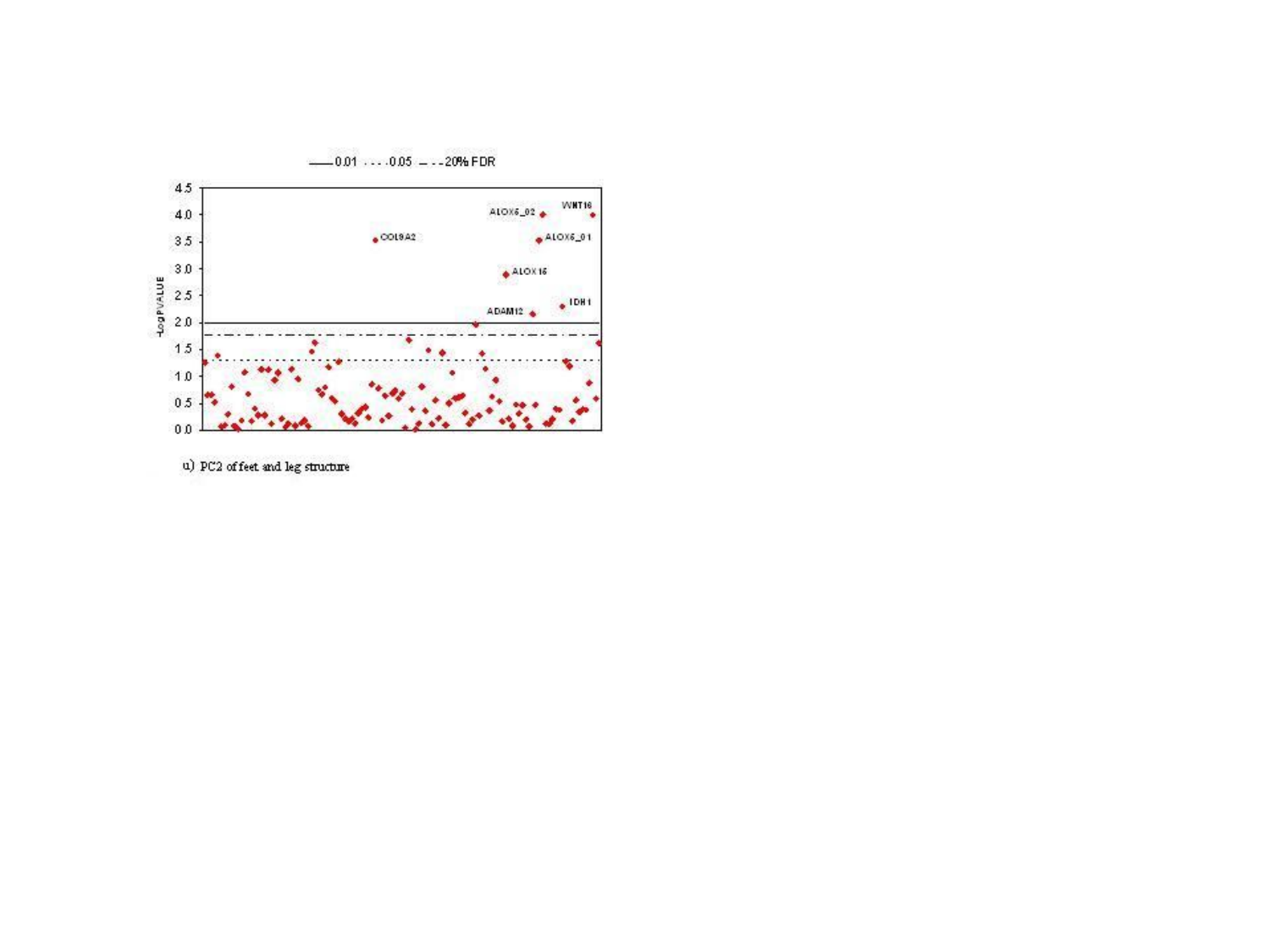

Supplement: Additional File 5 — Appendix Four. Association analyses results of single SNP markers with body conformation, feet and leg structure traits and principal factors in two commercially available breeding female lines (The x-axis indicates individual SNPs distributed along with pig chromosomes and y-axis indicates -log (P-value). SNPs with P < 0.001 and being under the 20% threshold of FDR are labeled. a) overall leg action; b) body depth; c) body length; d) body width; e) hip structure; f) rib shape; g) top line; h) front leg buck knee; i) front foot size; j) front pastern posture; k) front leg turned in/out; l) front uneven toes; m) rear foot size; n) rear pastern posture; o) rear leg turned in/out; p) rear uneven toes; q) rear weak leg; r) PC1 of body conformation traits; s) PC2 of body conformation traits; t) PC1 of feet and leg structure; u) PC2 of feet and leg structure). [file 1297-9686-41-14-S5.ppt]
