# Supplementary material for: Large-scale association study for structural soundness and leg locomotion traits in the pig
Source: Genet Sel Evol. 2009 Jan 21;41(1):14. doi: 10.1186/1297-9686-41-14 (PMC2657774; doi:10.1186/1297-9686-41-14)
Supplement: Additional File 2 — Table Two. The characteristics of the analyzed SNPs (SNPs were sorted by their chromosomal locations). [file 1297-9686-41-14-S2.doc]

Table 2. The characteristics of the analyzed SNPs (SNPs were sorted by their chromosomal locations).

| **Gene** | **Full name** | **dbSNP No.** | **SSC** | **SNP type** | **SNP location** | **Amino Acid change** | **No. animals** | **MAF** | **H-W P-value** |
| --- | --- | --- | --- | --- | --- | --- | --- | --- | --- |
| *NPP1* | Ectonucleotide pyrophosphatase 1 | ss86352339 | 1 | G/T | exon10 | Synonymous | 2023 | 0.3030 | 0.8910 |
| *COL9A1* | Collagen, type IX, alpha 1 | ss86352089 | 1 | A/C | intron4 |  | 2018 | 0.3957 | 0.7770 |
|  |  | ss86352090 | 1 | T/G | exon36 | TCA(Ser)→GCA (Ala) | 2052 | 0.4189 | 0.7849 |
| *MC4R* | Melanocortin 4 receptor | ss86352451 | 1 | A/G | exon1 | AAT(Asn)→GAT (Asp) | 2038 | 0.4605 | 0.3189 |
| *ESR2* | Estrogen receptor 2 | ss86352376 | 1 | A/G | exon5 | GUG(Val)→AUG (Met) | 1967 | 0.3042 | 0.8401 |
| *PAPPA* | Pregnancy-associated plasma protein A | ss86352425 | 1 | A/G | exon2 | Synonymous | 1946 | 0.2130 | 0.4744 |
| *FBN1* | Fibrillin 1 | ss86352269 | 1 | A/G | exon57 | GTG(Val)→ATG (Met) | 2026 | 0.0933 | 0.0944 |
| *CILP* | Cartilage intermediate layer protein | ss86352271 | 1 | C/T | exon7 | Synonymous | 2049 | 0.1240 | 0.0800 |
| *NFATC1* | Nuclear factor of activated T-cells, calcineurin-dependent 1 | ss86352275 | 1 | C/T | unknown |  | 2046 | 0.1950 | < 0.0001 |
| *TRAF6* | TNF receptor associated factor 6 | ss86352262 | 2 | A/T | intron4 |  | 2016 | 0.2269 | 0.7201 |
| *CALCA* | Calcitonin-related polypeptide alpha | ss86352250 | 2 | C/G | intron2 |  | 1795 | 0.0905 | < 0.0001 |
| *ADAMTS2* | ADAM metallopeptidase with thrombospondin type 1 motif, 2 | ss86352454 | 2 | C/T | exon21 | Synonymous | 2051 | 0.1390 | 0.0323 |
| *GR* | Glucocorticoid receptor | ss86352431 | 2 | A/G | exon2 | Synonymous | 1678 | 0.2589 | 0.1437 |
| *COL23A1* | Collagen, type XXIII, alpha 1 | ss86352457 | 2 | A/C | intron2 |  | 2022 | 0.2593 | 0.0002 |
| *TGFB1* | Transforming growth factor beta1 | ss86352468 | 2 | C/T | intron9 |  | 2052 | 0.2858 | 0.1202 |
| *NST* | Nucleotide sugar transporter | ss86352427 | 2 | C/T | 5'UTR |  | 2054 | 0.1526 | 0.1311 |
| *CART* | Cocaine and amphetamine regulated transcript | ss86352336 | 2 | G/T | intron2 |  | 1995 | 0.2689 | 0.5906 |
| *ALX4* | Aristaless-like homeobox 4 | ss86352378 | 2 | A/G | exon1 | Synonymous | 1941 | 0.2199 | 0.0907 |
| *SLC22A5* | Solute carrier family 22, member 5 | ss86352415 | 2 | C/G | intron9 |  | 2020 | 0.3540 | 0.9937 |
| *APIP* | APAF1 interacting protein | ss86352455 | 2 | A/G | intron4 |  | 2021 | 0.2412 | 0.1588 |
|  |  | ss86352456 | 2 | C/T | intron3 |  | 2002 | 0.1648 | 0.0441 |
| *FBN3* | Fibrillin 3 | ss86352458 | 2 | A/G | intron43 |  | 2021 | 0.1402 | 0.0241 |
| *LIN7C* | Lin-7 homolog C | ss86352462 | 2 | A/G | exon2 | Synonymous | 2018 | 0.1001 | 0.4261 |
| *IL13* | Interleukin 13 | ss86352495 | 2 | A/G | intron3 |  | 1943 | 0.4390 | 0.4883 |
| *HSD17B12* | Hydroxysteroid (17-beta) dehydrogenase 12 | ss86352497 | 2 | C/T | intron10 |  | 2020 | 0.1784 | 0.5104 |
| *ADAMTS19* | ADAM metallopeptidase with thrombospondin type 1 motif, 19 | ss86352502 | 2 | C/T | intron9 |  | 2022 | 0.4406 | 0.4916 |
| *FGFR4* | Fibroblast growth factor receptor 4 | ss86352507 | 2 | G/T | intron9 |  | 2022 | 0.3236 | 0.3528 |
| *INSL3* | Insulin-like 3 | ss86352510 | 2 | A/C | 5’UTR |  | 2022 | 0.2366 | 0.0127 |
| *LRP5* | Low density lipoprotein receptor-related protein 5 | ss86352157 | 2 | C/T | intron11 |  | 1952 | 0.1483 | 0.4195 |
|  |  | ss86352158 | 2 | A/C | exon23 | Synonymous | 2054 | 0.1273 | 0.8882 |
|  |  | ss86352159 | 2 | A/G | exon23 | Synonymous | 2015 | 0.1199 | 0.3917 |
| *IL1RA* | Interleukin 1 receptor antagonist | ss86352173 | 3 | C/T | exon6 | Synonymous | 2051 | 0.2450 | 0.0920 |
| *MATN3* | Matrilin 3 | ss86352213 | 3 | A/G | exon2 | Synonymous | 1927 | 0.1559 | 0.0035 |
|  |  | ss86352214 | 3 | A/G | intron2 |  | 2026 | 0.3778 | 0.7220 |
| *OPG* | Osteoprotegerin | ss86352133 | 4 | A/G | exon4 | Synonymous | 2054 | 0.4160 | 0.0610 |
|  |  | ss86352136 | 4 | C/T | intron4 |  | 2017 | 0.3679 | 0.5681 |
| *CSF1* | Colony stimulating factor 1 | ss86352345 | 4 | C/G | exon6 | ACC(Thr)→AGC(Ser) | 1943 | 0.2009 | 0.4386 |
| *WARS2* | Tryptophanyl tRNA synthetase 2 | ss86352416 | 4 | A/G | exon2 |  | 2055 | 0.3540 | 0.4279 |
| *WNT7B* | Wingless related MMTV integration site 7B | ss86352476 | 5 | A/G | intron1 |  | 2020 | 0.1755 | 0.9037 |
| *WNT10B* | Wingless related MMTV integration site 10 B | ss86352479 | 5 | A/G | intron4 |  | 1933 | 0.3826 | 0.0014 |
| *SP7* | Sp7 transcription factor | ss86352256 | 5 | C/T | exon1 | Synonymous | 1951 | 0.1251 | 0.0491 |
| *PTHLH* | Parathormone like peptide | ss86352176 | 5 | C/T | intron2 |  | 1948 | 0.1684 | 0.3976 |
| *IFN* | Interferon gamma | ss86352488 | 5 | C/T | intron1 |  | 2022 | 0.3165 | 0.6383 |
| *VDR* | Vitamin D receptor | ss86352119 | 5 | C/T | exon9 | Synonymous | 2051 | 0.2072 | 0.0104 |
| *COL2A1* | Collagen, type II, alpha 1 | ss86352100 | 5 | C/T | intron32 |  | 1954 | 0.3133 | 0.1752 |
|  |  | ss86352103 | 5 | T/G | intron53 |  | 1962 | 0.1027 | 0.1937 |
| *MMP2* | Matrix Metalloproteinases | ss86352465 | 6 | C/G | 5'UTR |  | 2023 | 0.1935 | 0.8014 |
| *MTHFR* | Methylene Tetrahydrofolate Reductase | ss86352182 | 6 | A/G | 5'UTR |  | 2025 | 0.4333 | 0.0986 |
|  |  | ss86352185 | 6 | A/G | exon2 | Synonymous | 2009 | 0.4333 | 0.1184 |
| *CMP* | Cartilage matrix protein | ss86352202 | 6 | C/T | exon7 | Synonymous | 2051 | 0.7345 | 0.7818 |
|  |  | ss86352203 | 6 | A/G | intron7 |  | 1871 | 0.8220 | 0.0899 |
| *COL9A2* | Collagen, type IX, alpha 2 | ss86352243 | 6 | C/T | intron31 |  | 1991 | 0.4771 | 0.8779 |
| *APOE* | Apolipoprotein E | ss86352253 | 6 | C/T | intron2 |  | 2042 | 0.2060 | 0.7260 |
| *PKN2* | Protein kinase N2 | ss86352300 | 6 | A/T | intron14 |  | 2017 | 0.2206 | 0.4509 |
| *BMP8* | Bone morphogenetic protein 8 | ss86352165 | 6 | A/G | intron2 |  | 1962 | 0.2541 | <0.0001 |
| *TNFα* | Tumor necrosis factor α | ss86352149 | 7 | G/T | intron1 |  | 1963 | 0.1824 | <0.0001 |
|  |  | ss86352152 | 7 | A/G | 3'UTR |  | 2053 | 0.4725 | 0.4100 |
| *COL11A2* | Collagen, type XI, alpha 2 | ss86352190 | 7 | A/T | intron60 |  | 1957 | 0.5156 | <0.0001 |
| *ACAN* | Aggrecan | ss86352211 | 7 | A/G | exon8 | GGG (Gly)→AGG (Arg) | 2039 | 0.8865 | 0.0063 |
| *HSP90* | 90-kDa heat shock protein | ss86352357 | 7 | A/T | intron5 |  | 1954 | 0.2988 | <0.0001 |
|  |  | ss86352367 | 7 | C/T | intron6 |  | 2011 | 0.0783 | 0.0001 |
| *WARS* | Tryptophanyl-tRNA synthetase | ss86352417 | 7 | C/T | intron5 |  | 2017 | 0.4199 | 0.8639 |
| *VDBP* | Vitamin D binding protein | ss86352124 | 8 | C/T | intron4 |  | 2010 | 0.3371 | 0.7372 |
|  |  | ss86352125 | 8 | A/G | intron4 |  | 2026 | 0.4472 | 0.7924 |
| *Nocturnin* | Nocturnin | ss86352276 | 8 | C/T | exon8 | ACA (Thr)→ATA (Ile) | 2053 | 0.2857 | 0.7091 |
| *FGF2* | Fibroblast growth factor 2 | ss86352327 | 8 | C/T | exon1 | ACC (Thr)→ATC(Ile) | 2053 | 0.4415 | 0.1718 |
| *BMPR1B* | Bone morphogenic protein receptor 1 | ss86352480 | 8 | G/T | intron7 |  | 2023 | 0.2173 | 0.6489 |
|  |  | ss86352482 | 8 | C/G | intron8 |  | 2020 | 0.1228 | 0.9263 |
|  |  | ss86352485 | 8 | A/G | intron4 |  | 2016 | 0.4965 | 0.0407 |
| *OPN* | Osteopontin | ss86352322 | 8 | A/G | 5'UTR |  | 1972 | 0.2147 | 0.5979 |
| *MEPE* | Matrix extracellular phosphoglycoprotein with ASARM motif | ss86352394 | 8 | A/G | exon 5 | Synonymous | 2047 | 0.2137 | 0.6447 |
|  |  | ss86352395 | 8 | C/T | exon 5 | Synonymous | 2059 | 0.3441 | 0.4659 |
| *GNRHR* | Gonadotropin-releasing hormone receptor | ss86352460 | 8 | G/T | 5'UTR |  | 2035 | 0.2054 | 0.1402 |
| *COL1A2* | Collagen, type I, alpha 2 | ss86352086 | 9 | C/T | intron13 |  | 1943 | 0.2205 | 0.2619 |
|  |  | ss86352087 | 9 | C/T | exon18 | Synonymous | 2052 | 0.2339 | 0.9724 |
| *CALCR* | Calcitonin receptor | ss86352109 | 9 | A/G | intron9 |  | 2052 | 0.2420 | 0.5367 |
|  |  | ss86352112 | 9 | C/G | 3'UTR |  | 2033 | 0.2425 | 0.6680 |
|  |  | ss86352113 | 9 | C/T | 3'UTR |  | 2051 | 0.2423 | 0.5135 |
|  |  | ss86352114 | 9 | A/C | 3'UTR |  | 2019 | 0.3900 | 0.4453 |
| *IL6* | Interleukin 6 | ss86352128 | 9 | C/T | intron2 |  | 2004 | 0.1826 | 0.1857 |
| *SFRP4* | Secreted frizzled-related protein 4 | ss86352472 | 9 | A/G | intron3 |  | 2023 | 0.0536 | 0.7197 |
| *KLOTHO* | Klotho | ss86352189 | 11 | C/T | exon4 | Synonymous | 1885 | 0.4440 | 0.0010 |
| *LRCH1* | Leucine-rich repeats and calponin homology domain containing 1 | ss86352234 | 11 | C/T | intron5 |  | 2026 | 0.3798 | 0.5886 |
| *RANKL* | Tumor necrosis factor (ligand) superfamily, member 11 | ss86352129 | 11 | A/G | intron4 |  | 1685 | 0.3427 | 0.7396 |
|  |  | ss86352131 | 11 | C/T | 3'UTR |  | 2053 | 0.4101 | 0.8790 |
| *VTN* | Vitronectin | ss86352325 | 12 | A/G | Intron3 |  | 1862 | 0.2102 | 0.2458 |
| *SYNGR2* | Synaptogyrin 2 | ss86352466 | 12 | C/T | intron2 |  | 2050 | 0.3851 | 0.2609 |
|  |  | ss86352467 | 12 | C/T | exon2 |  | 2048 | 0.2375 | 0.3559 |
| *TIMP2* | TIMP metallopeptidase inhibitor 2 | ss86352469 | 12 | C/T | intron3 |  | 2033 | 0.0380 | 0.9598 |
| *DKFZ* | Hypothetical protein DKFZp434P0316 | ss86352506 | 12 | A/G | unknown |  | 2022 | 0.0702 | 0.3024 |
| *ALOX15* | Arachidonate 15-lipoxygenase | ss86352146 | 12 | A/C | intron7 |  | 1758 | 0.3979 | <0.0001 |
| *CTNNB1* | Catenin (cadherin-associated protein), beta 1 | ss86352341 | 13 | C/G | intron9 |  | 2050 | 0.4439 | 0.9252 |
| *PTHR1* | Parathormone receptor 1 | ss86352174 | 13 | A/G | intron12 |  | 2048 | 0.4480 | 0.0700 |
| *PPARG* | Peroxisome proliferator-activated receptor gamma | ss86352388 | 13 | A/G | 5'UTR |  | 1875 | 0.1515 | 0.7171 |
| *OXTR* | Oxytocin receptor | ss86352270 | 13 | G/T | exon3 | Synonymous | 2013 | 0.2707 | 0.0193 |
| *CASR* | Calcium sensing receptor | ss86352179 | 13 | C/T | intron6 |  | 2040 | 0.3966 | 0.1432 |
| *COL8A1* | Collagen, type VIII, alpha 1 | ss86352088 | 13 | C/T | exon3 | CTT(Leu)→TTT(Phe) | 2011 | 0.4316 | 0.0644 |
| *GGT1* | Gamma glutamyl transferase 1 | ss86352433 | 14 | C/G | intron10 |  | 2033 | 0.4085 | 0.8357 |
| *ADAM12* | ADAM metallopeptidase domain 12 | ss86352230 | 14 | C/T | intron20 |  | 2025 | 0.2926 | 0.0740 |
| *LIF* | Leukemia inhibitory factor | ss86352245 | 14 | C/T | 3'UTR |  | 1938 | 0.3885 | 0.0331 |
| *ALOX5* | Arachidonate 5-lipoxygenase | ss86352443 | 14 | G/T | intron4 |  | 2051 | 0.4805 | 0.1436 |
|  |  | ss86352445 | 14 | C/G | intron4 |  | 1688 | 0.4449 | 0.2837 |
| *FGFR1* | Fibroblast growth factor receptor 1 | ss86352342 | 14 | C/G | exon13 | Synonymous | 1937 | 0.0913 | 0.2951 |
|  |  | ss86352344 | 14 | A/G | exon9 | Synonymous | 1908 | 0.0936 | 0.7255 |
| *NRP2* | Neurophilin 2 | ss86352299 | 15 | C/T | intron2 |  | 1986 | 0.2951 | <0.0001 |
| *IGFBP2* | Insulin like growth factor binding protein 2 | ss86352411 | 15 | A/G | intron2 |  | 2046 | 0.1444 | 0.9541 |
| *IHH* | Indian hedgehog | ss86352349 | 15 | A/G | exon3 | Synonymous | 2019 | 0.4049 | 0.2681 |
| *IDH1* | Isocitrate dehydrogenase 1 | ss86352295 | 15 | C/T | exon4 | CGT(Arg)→TGT (Cys) | 1924 | 0.1076 | 0.0025 |
| *IGFBP5* | Insulin like growth factor binding protein 5 | ss86352413 | 15 | A/C | intron2 |  | 2053 | 0.1203 | 0.7203 |
| *ANKH* | Ankylosis homolog | ss86352216 | 16 | C/T | intron11 |  | 2024 | 0.3740 | 0.4404 |
| *SPARC* | Secreted protein, acidic, cysteine-rich | ss86352238 | 16 | C/T | 3'UTR |  | 1927 | 0.1946 | 0.4653 |
| *MMP9* | Matrix metallopeptidase 9 | ss86352323 | 17 | C/G | intron6 |  | 2049 | 0.4161 | 0.0599 |
| *BMP7* | Bone morphogenic protein 7 | ss86352286 | 17 | A/G | intron3 |  | 1824 | 0.2385 | 0.0007 |
| *GNAS* | GNAS complex locus | ss86352247 | 17 | C/G | intron8 |  | 2028 | 0.3964 | 0.7626 |
|  |  | ss86352249 | 17 | A/T | intron8 |  | 2012 | 0.3966 | 0.8161 |
| *ENOS* | Endothelial nitric oxide synthase 3 | ss86352296 | 18 | A/T | intron12 |  | 2024 | 0.1292 | 0.0001 |
| *WNT16* | Wingless-type MMTV integration site family, member 16 | ss86352423 | 18 | C/T | intron1 |  | 2052 | 0.3540 | 0.4279 |
| *IGFBP1* | Insulin like growth factor binding protein 1 | ss86352409 | 18 | C/T | intron2 |  | 2021 | 0.4057 | 0.2201 |
| *WNT2* | Wingless-type MMTV integration site family member 2 | ss86352422 | 18 | C/T | intron4 |  | 2027 | 0.3709 | 0.6332 |
