# Supplementary material for: Large-scale association study for structural soundness and leg locomotion traits in the pig
Source: Genet Sel Evol. 2009 Jan 21;41(1):14. doi: 10.1186/1297-9686-41-14 (PMC2657774; doi:10.1186/1297-9686-41-14)
Supplement: Additional File 3 — Appendix Two. The phenotypic correlations between the 17 analyzed traits of body conformation, feet and leg structure and overall leg action. [file 1297-9686-41-14-S3.doc]

Appendix 2. The phenotypic correlations between the 17 analyzed traits of body conformation, feet and leg structure and overall leg action (The P-values are within the upper right-hand triangle and the correlation coefficients are within the lower left-hand triangle).

| **Trait^*^** | **BL** | **BD** | **TL** | **HS** | **RS** | **BW** | **RT** | **WR** | **RP** | **RF** | **RU** | **FT** | **BK** | **FP** | **FF** | **FU** | **LA** |
| --- | --- | --- | --- | --- | --- | --- | --- | --- | --- | --- | --- | --- | --- | --- | --- | --- | --- |
| BL | 1 | <0.0001 | 0.0098 | <0.0001 | <0.0001 | 0.0016 | 0.0020 | 0.0025 | <0.0001 | 0.0215 | 0.0138 | 0.6343 | <0.0001 | <0.0001 | <0.0001 | 0.2448 | <0.0001 |
| BD | 0.13 | 1 | 0.3687 | <0.0001 | <0.0001 | <0.0001 | 0.0008 | 0.0771 | 0.0004 | <0.0001 | 0.3564 | 0.5705 | 0.5570 | <0.0001 | <0.0001 | 0.0261 | 0.0191 |
| TL | 0.06 | 0.02 | 1 | <0.0001 | 0.0067 | 0.0102 | 0.0244 | 0.0005 | 0.0024 | 0.7868 | 0.3954 | 0.8721 | 0.0167 | 0.0003 | 0.6941 | 0.0027 | 0.0014 |
| HS | 0.12 | -0.16 | 0.24 | 1 | 0.5034 | 0.0315 | <0.0001 | 0.0192 | <0.0001 | 0.0002 | 0.0270 | 0.0298 | <0.0001 | <0.0001 | <0.0001 | 0.0011 | <0.0001 |
| RS | 0.25 | 0.50 | 0.06 | 0.02 | 1 | <0.0001 | 0.0001 | 0.2422 | <0.0001 | <0.0001 | 0.2953 | 0.3136 | 0.0003 | <0.0001 | <0.0001 | 0.2426 | 0.0150 |
| BW | -0.07 | -0.52 | -0.06 | -0.05 | -0.46 | 1 | <0.0001 | 0.1236 | <0.0001 | <0.0001 | 0.3017 | 0.4773 | 0.0002 | <0.0001 | <0.0001 | 0.5659 | 0.0797 |
| RT | 0.07 | 0.08 | 0.05 | 0.10 | 0.09 | -0.11 | 1 | <0.0001 | 0.0079 | 0.6416 | 0.2728 | 0.9696 | <0.0001 | <0.0001 | 0.1928 | 0.3303 | <0.0001 |
| WR | 0.07 | 0.04 | 0.08 | 0.05 | 0.03 | -0.04 | 0.11 | 1 | 0.0018 | 0.0337 | 0.4111 | 0.6834 | 0.0016 | <0.0001 | 0.4124 | 0.0994 | <0.0001 |
| RP | 0.12 | 0.08 | 0.07 | 0.13 | 0.10 | -0.11 | 0.06 | 0.07 | 1 | <0.0001 | 0.0519 | <0.0001 | <0.0001 | <0.0001 | 0.0528 | 0.0930 | <0.0001 |
| RF | -0.05 | 0.11 | -0.01 | 0.08 | 0.10 | -0.20 | 0.01 | 0.05 | 0.30 | 1 | 0.2070 | 0.2278 | 0.0001 | <0.0001 | <0.0001 | 0.0003 | <0.0001 |
| RU | 0.06 | -0.02 | -0.02 | 0.05 | -0.03 | -0.02 | -0.02 | 0.02 | 0.05 | 0.03 | 1 | 0.1358 | <0.0001 | <0.0001 | 0.3496 | <0.0001 | <0.0001 |
| FT | -0.01 | 0.01 | 0.003 | -0.05 | -0.02 | 0.02 | -0.002 | 0.01 | -0.09 | -0.03 | -0.03 | 1 | 0.5317 | 0.0008 | 0.9677 | 0.2981 | 0.2001 |
| BK | 0.16 | 0.01 | 0.05 | 0.26 | 0.08 | -0.08 | 0.11 | 0.07 | 0.11 | 0.09 | 0.10 | -0.01 | 1 | <0.0001 | <0.0001 | 0.2699 | <0.0001 |
| FP | 0.20 | 0.20 | 0.08 | 0.22 | 0.18 | -0.16 | 0.13 | 0.12 | 0.29 | 0.17 | 0.12 | -0.08 | 0.27 | 1 | <0.0001 | <0.0001 | <0.0001 |
| FF | -0.11 | 0.23 | -0.01 | -0.13 | 0.14 | -0.21 | 0.03 | -0.02 | 0.04 | 0.32 | -0.02 | -0.0003 | -0.11 | 0.19 | 1 | <0.0001 | 0.0847 |
| FU | -0.03 | -0.05 | 0.07 | 0.07 | -0.03 | -0.01 | 0.02 | 0.04 | 0.04 | 0.08 | 0.15 | -0.02 | 0.03 | 0.10 | 0.10 | 1 | <0.0001 |
| LA | 0.16 | 0.05 | 0.07 | 0.18 | 0.06 | -0.04 | 0.16 | 0.30 | 0.19 | 0.11 | 0.11 | 0.03 | 0.31 | 0.39 | 0.04 | 0.10 | 1 |

^*^BL, body length; BD, body depth; TL, top line; HS, hip structure; RS, rib shape; BD, body width; RT, rear leg turned in/out; WR, rear weak leg; RP, rear pastern posture; RF, rear foot size; RU, rear uneven toes; FT, front leg turned in/out; BK, front leg buck knee; FP, front pastern posture; FF, front foot size; FU, front uneven toes; LA, overall leg action.
