# Supplementary material for: Large-scale association study for structural soundness and leg locomotion traits in the pig
Source: Genet Sel Evol. 2009 Jan 21;41(1):14. doi: 10.1186/1297-9686-41-14 (PMC2657774; doi:10.1186/1297-9686-41-14)
Supplement: Additional File 4 — Appendix Three. The eigenvalues and eigenvectors of principal component (PC) analysis on the 17 analyzed traits. [file 1297-9686-41-14-S4.doc]

Appendix 3. The eigenvalues and eigenvectors of principal component (PC) analyses on the 17 analyzed traits.

| **Group** | **Trait^*^** | **PC1** | **PC2** | **PC3** |
| --- | --- | --- | --- | --- |
| Body conformation | Eigenvalue | 2.055 | 1.305 | 0.946 |
|  | Proportion (%) | 0.343 | 0.217 | 0.157 |
|  | Cumulative (%) | 0.343 | 0.560 | 0.718 |
|  | BL | 0.246 | 0.306 | **0.849** |
|  | BD | **0.566** | -0.210 | -0.110 |
|  | BW | **-0.544** | 0.027 | 0.286 |
|  | RS | **0.564** | 0.020 | 0.084 |
|  | HS | -0.006 | **0.705** | -0.056 |
|  | TL | 0.086 | **0.604** | -0.417 |
| Feet and leg structure | Eigenvalue | 2.162 | 1.358 | 1.120 |
|  | Proportion (%) | 0.196 | 0.124 | 0.102 |
|  | Cumulative (%) | 0.196 | 0.320 | 0.422 |
|  | RT | 0.198 | -0.200 | 0.346 |
|  | WR | 0.254 | -0.278 | 0.247 |
|  | RP | **0.362** | 0.187 | 0.028 |
|  | RF | 0.314 | **0.510** | 0.106 |
|  | RU | 0.163 | -0.076 | **-0.675** |
|  | FT | -0.067 | -0.109 | 0.279 |
|  | BK | **0.334** | -0.346 | -0.094 |
|  | FP | **0.489** | 0.012 | -0.017 |
|  | FF | 0.179 | **0.595** | 0.184 |
|  | FU | 0.170 | 0.129 | **-0.471** |
|  | LA | **0.471** | -0.279 | 0.086 |

^*^BL, body length; BD, body depth; TL, top line; HS, hip structure; RS, rib shape; BD, body width; RT, rear leg turned in/out; WR, rear weak leg; RP, rear pastern posture; RF, rear foot size; RU, rear uneven toes; FT, front leg turned in/out; BK, front leg buck knee; FP, front pastern posture; FF, front foot size; FU, front uneven toes; LA, overall leg action.
