# Supplementary material for: Large-scale association study for structural soundness and leg locomotion traits in the pig
Source: Genet Sel Evol. 2009 Jan 21;41(1):14. doi: 10.1186/1297-9686-41-14 (PMC2657774; doi:10.1186/1297-9686-41-14)
Supplement: Additional File 6 — Table Three. The list of significant SNPs for the 17 analyzed traits of body conformation, feet and leg structure and overall leg action. [file 1297-9686-41-14-S6.doc]

Table 3. The list of significant SNPs for the 17 analyzed traits of body conformation, feet and leg structure and overall leg action.

| **Trait** | **Very highly significant (P<0.001)** | **Highly significant (P<0.01)** | **Significant (P<0.05)** |
| --- | --- | --- | --- |
| Body length | *APOE* | *CART, COL9A1* (ss86352089, ss86352090)*, OPG* (ss86352133)*, COL11A2* | *PAPPA, CILP, TGFB1, LRP5* (ss86352157, ss86352158)*, OPG* (ss86352136)*, WARS2, MTHFR* (ss86352182, ss86352185)*, VDBP* (ss86352124, ss86352125)*, COL1A2 (*ss86352087)*, CALCR* (ss86352112, ss86352113), *SFRP4, DKFZ, OXTR, FGFR1* (ss86352342)*, GNAS* (ss86352247, ss86352249)*, WNT2* |
| Body depth | *ADAMTS2* | *COL9A1* (ss86352089, ss86352090) | *NPP1, MC4R, PAPPA, CALCA, NST, HSD17B12, INSL3, COL2A1* (ss86352103)*, APOE, COL11A2, VTN, SYNGR2* (ss86352467), *ANKH, GNAS* (ss86352247, ss86352249) |
| Body width | *COL9A1* (ss86352089, ss86352090), *VDBP*  (ss86352125) | *NST, CART, VDBP* (ss86352124), *GNRHR, RANKL* (ss86352131), *DKFZ, PPARG, IHH* | *ADAMTS2, SLC22A5, FGFR4, INSL3, OPG* (ss86352136), *PTHLH, APOE, TNFα* (ss86352152), *HSP90* (ss86352357), *ALOX15, SPARC* |
| Top Line | *FBN1, BMP8* | *WNT16* | *CSF1, COL9A2, OXTR, WNT2* |
| Hip structure | *COL1A2* (ss86352086, ss86352087)*, CALCR* (ss86352109, ss86352112, ss86352113) | *CART, ADATS19, CALCR* (ss86352114)*, PTHR1, OXTR* | *FBN1, CALCA, COL23A1, ALX4, MMP2, TNFα* (ss86352149)*, HSP90* (ss86352357)*, VDBP* (ss86352124, ss86352125), *RANKL* (ss86352129, ss86352131)*, VTN, ADAM12* |
| Rib shape | *-* | *PAPPA, HSP90* (ss86352357)*, VDBP* (ss86352125) | *COL9A1* (ss86352089), *ALX4, APIP* (ss86352455), *HSD17B12, INSL3, WARS2, APOE, VDBP* (ss86352124)*, CALCR* (ss86352114)*, RANKL* (ss86352129, ss86352131)*, SYNGR2* (ss86352466)*, PPARG, SPARC, WNT16* |
| Front legs turned in/out | *APIP* (ss86352456)*, LRCH1* | *-* | *MC4R, ESR2, TRAF6, APIP* (ss86352455)*, LIN7C, SP7, TNFα* (ss86352152)*, HSP90,* (ss86352367)*, MEPE* (ss86352395)*, COL1A2* (ss86352087), *CALCR* (ss86352109, ss86352113)*, PTHR1, IGFBP1* |
| Rear legs turned in/out | *MC4R, ESR2, WARS2* | *FGFR4, COL9A2, VTN, WNT16* | *CALCA, CSF1, APOE, KLOTHO, SYNGR2* (ss86352467)*, ALOX5* (ss86352445)*, SPARC* |
| Front pastern posture | *BMP8, BMPR1B* (ss86352480)*, OXTR* | *COL2A1,* (ss86352100)*, BMPR1B* (ss86352482)*, CALCR* (ss86352114)*, CASR* | *NST, CART, COL2A1* (ss86352103)*, COL9A2, APOE, COL1A2* (ss86352086, ss86352087), *CALCR* (ss86352109, ss86352112, ss86352113)*, PTHR1, LIF, SPARC* |
| Rear pastern posture | *FGFR4* | *OPG* (ss86352136)*, BMPR1B* (ss86352482)*, COL1A2* (ss86352087)*, CALCR* (ss86352112, ss86352113, ss86352114)*, SRFP4, OXTR, MMP9* | *TNFα* (ss86352149)*, WARS, FGF2, CALCR* (ss86352109)*, LRCH1, PTHR1, CASR, ADAM12* |
| Front foot size | *LRP5* (ss86352157) | *MATN3* (ss86352214)*, COL9A2, ALOX15, WNT16* | *COL23A1, WNT7B, COL2A1* (ss86352100)*, BMP8, OXTR, ADAM12, ANKH, MMP9* |
| Rear foot size | *-* | *MATN3* (ss86352214)*, RANKL* (ss86352129)*, ALOX15* | *NFACT1, FGFR4, OPG* (ss86352133)*, WNT7B, MMP2, NOCT1, RANKL* (ss86352131)*, ALOX5* (ss86352443, ss86352444)*, ENOS, WNT16* |
| Front uneven toes | *OPG* (ss86352133) | *PAPPA, OPG* (ss86352136)*, COL2A1* (ss86352100)*, MEPE* (ss86352394) | *APIP* (ss86352455)*, VDR, COL1A2* (ss86352087)*, CALCR* (ss86352112, ss86352113)*, ALOX15, PPARG, IGFBP1* |
| Rear uneven toes | *PPARG* | *-* | *PAPPA, MATN3* (ss86352213)*, OPG* (ss86352136)*, WARS2, WNT10B, COL9A2, BMP8, BMPR1B* (ss86352485) |
| Buck knee | *-* | *CALCA, GR, WNT16* | *CILP, ALX4, MATN3* (ss86352214)*, FGF2, RANKL* (ss86352131)*, ALOX15, CTNNB1* |
| Weak rear legs | *-* | *IHH, GNAS* (ss86352249) | *APIP* (ss86352455), *FBN3, CMP* (ss86352202), *BMP8, PTHR1, ANKH, MMP9, GNAS* (ss86352247) |
| Overall leg action | *APOE* | *MTHFR* (ss86352182, ss86352185)*, GNRHR, CALCR* (ss86352114)*, WNT16, IHH* | *ESR2, GR, NST, CART, OPG* (ss86352133, ss86352136), *WNT7B, BMP8, COL1A2* (ss86352086, ss86352087), *CALCR* (ss86352109, ss86352112, ss86352113), *LRCH1, ANKH* |
| Body conformation principal component 1 | *COL9A1* (ss86352089, ss86352090)*, PAPPA, VDBP* (ss86352125)*, DKFZ* | *NST, APOE, VDBP* (ss86352124)*, PPARG* | *NPP1, ADAMTS2, CART, INSL3, PTHRI, HSP90* (ss86352357), *LRCH1, WNT16, SPARC* |
| Body conformation principal component 2 | *FBN1, COL1A2* (ss86352087)*, CALCR* (ss86352109, ss86352112, ss86352113)*, OXTR* | *CALCA, ADAMTS19, MATN3* (ss86352214)*, COL1A2* (ss86352086) | *MC4R, NFACT1, SLC22A5, WNT7B, HSP90* (ss86352367), *WARS, VTN, PTHR1, SPARC* |
| Feet and leg structure principal component 1 | *CALCR* (ss86352113, ss86352114)*, OXTR* | *OPG* (ss86352136)*, BMP8, NOCT1, COL1A2* (ss86352086, ss86352087)*, CALCR* (ss86352109, (ss86352112)*, PTHRI* | *ESR2, WNT7B, WARS, VDBP* (ss86352124), *RANKL* (ss86352131), *IHH* |
| Feet and leg structure principal component 2 | *COL9A2, ALOX5* (ss86352443, ss86352445)*, WNT16* | *ADAM12, ALOX15, IDH1* | *ESR2, MATN3* (ss86352213, ss86352214), *WARS, BMPR1B* (ss86352482), *MEPE* (ss86352395), *KLOTHO, RANKL* (ss86352129), *WNT2* |

(The dbSNP numbers were listed for genes with multiple SNPs.)
