# Supplementary material for: Large-scale association study for structural soundness and leg locomotion traits in the pig
Source: Genet Sel Evol. 2009 Jan 21;41(1):14. doi: 10.1186/1297-9686-41-14 (PMC2657774; doi:10.1186/1297-9686-41-14)
Supplement: Additional File 7 — Table Four. The association analyses between putative haplotypes of CALCR and COL1A2 in SSC9, and the individual traits as well as principal components. [file 1297-9686-41-14-S7.doc]

Table 4. The association analyses between putative haplotypes of *CALCR* and *COL1A2* in SSC9, and the individual traits as well as principal components.

| Trait ^*^ | LSM ± SE^**^ for different copies of haplotype 1 (-ACGACC-) | | | P value | LSM ± SE^**^ for different copies of haplotype 2  (-CTCGTT-) | | | P value | LSM ± SE^**^ for different copies of haplotype 3 (-CCGACC-) | | | P value |
| --- | --- | --- | --- | --- | --- | --- | --- | --- | --- | --- | --- | --- |
|  | 0 | 1 | 2 |  | 0 | 1 | 2 |  | 0 | 1 | 2 |  |
| Sample size | 275 (13.7%) | 973 (48.5%) | 758 (37.89%) |  | 1172 (58.4%) | 734 (36.6%) | 100 (5.0%) |  | 1462 (72.9%) | 499 (24.9%) | 45 (2.2%) |  |
| Overall leg action | 4.47 ± 0.11^a^ | 4.84± 0.07^b^ | 4.80 ± 0.07^b^ | 0.0092 | 4.77 ± 0.06 | 4.84 ± 0.08 | 4.39 ± 0.18 | 0.0643 | 4.81 ± 0.06 | 4.70 ± 0.08 | 4.35 ± 0.27 | 0.1455 |
| Hip structure | 4.50 ± 0.09^a^ | 4.33 ± 0.05^b^ | 4.18± 0.06^c^ | 0.0047 | 4.18 ± 0.05^a^ | 4.43 ± 0.06^b^ | 4.75 ± 0.15^c^ | 0.0001 | 4.32 ± 0.05 | 4.24 ± 0.07 | 4.24 ± 0.22 | 0.5480 |
| Rear pastern posture | 3.98 ± 0.08^a^ | 4.30 ± 0.05^b^ | 4.38 ± 0.05^b^ | 0.0001 | 4.36 ± 0.05^a^ | 4.24 ± 0.05^b^ | 3.83 ± 0.13^c^ | 0.0004 | 4.31 ± 0.04 | 4.26 ± 0.07 | 4.11 ± 0.20 | 0.5284 |
| Front pastern posture | 4.26 ± 0.10^a^ | 4.61 ± 0.06^b^ | 4.63 ± 0.06^b^ | 0.0026 | 4.58 ± 0.06^a^ | 4.62 ± 0.07^a^ | 4.12 ± 0.16^b^ | 0.0120 | 4.61 ± 0.05^a^ | 4.53 ± 0.08^a^ | 3.97 ± 0.24^b^ | 0.0263 |
| BC PC1 | -0.08 ± 0.08 | 0.14 ± 0.06 | 0.09 ± 0.06 | 0.0613 | 0.12 ± 0.05 | 0.09 ± 0.06 | -0.15 ± 0.14 | 0.1763 | 0.08 ± 0.05 | 0.10 ± 0.07 | 0.10 ± 0.20 | 0.9638 |
| BC PC2 | 0.07 ± 0.06 | 0.01 ± 0.04 | -0.02 ± 0.04 | 0.4334 | -0.05 ± 0.03^a^ | 0.04 ± 0.04^b^ | 0.36 ± 0.10 ^c^ | 0.0002 | 0.04 ± 0.03 | -0.10 ± 0.05 | -0.12 ± 0.15 | 0.0197 |
| FL PC1 | -0.23 ± 0.09^a^ | 0.14 ± 0.05^b^ | 0.14 ± 0.06^b^ | 0.0002 | 0.12 ± 0.05^a^ | 0.11 ± 0.06^a^ | -0.41 ± 0.14^b^ | 0.0012 | 0.11 ± 0.05 | 0.08 ± 0.07 | -0.32 ± 0.31 | 0.1164 |
| FL PC2 | -0.08 ± 0.07 | -0.03 ± 0.04 | -0.03 ± 0.05 | 0.7731 | 0.001 ± 0.04 | -0.08 ± 0.05 | -0.11 ± 0.11 | 0.2466 | -0.05 ± 0.04 | -0.01 ± 0.06 | 0.04 ± 0.17 | 0.5124 |

^*^ BC PC1, the first principal component of body conformation traits, describing body volume; BC PC2, the second principal component of body conformation traits, describing body side profile; FL PC1, the first principal component of feet and leg soundness traits, describing leg movement condition; FL PC2, the second principal component of feet and leg soundness traits, describing feet defects.

^**^ LSM ± SE, Least square means ± standard error for phenotypic scores. The values bearing a, b, and/or c differ significantly (P < 0.05) from each other.
